# Tender Thigh in a Patient With Crohn's Disease

**DOI:** 10.4103/1319-3767.43283

**Published:** 2008-10

**Authors:** Abdulwahed Al-Saeed, Ahmed Helmy, Hamad Al-Ashgar, Khalid Al-Kahtani

**Affiliations:** Department of Medicine, Section of Gastroenterology, King Faisal Specialist Hospital and Research Centre, Riyadh, Saudi Arabia

A 25 year-old-male was known to have fistulizing Crohn's disease complicated with prerectal, pararectal, and gluteal abscesses as well as sacroiliac osteomyelitis. He underwent status fistula repair (twice) and abscess drainage (twice). He presented to the emergency department with pain in the right upper thigh of one day's duration. The pain was continuous, of rapid onset, moderate in severity, pressure-like in nature, aggravated by walking, and was associated with fever, chills, rigor, and arthralgia. He also had a dull-aching abdominal pain, and frequent nonbloody diarrhea with mucus. He denied any vomiting, weight loss, or ocular pain or redness. A review of other systems did not reveal any remarkable results.

Clinically, he was conscious, oriented, had a temperature of 38.9° C, a pulse of 115 beats per minute, blood pressure of 88/50 mm Hg, and a respiratory rate of 18 breaths per minute. His chest, cardiovascular, and central nervous system examinations also did not reveal any remarkable results. His abdomen was soft, lax, but mildly tender below the umbilicus. Local examination of the site of pain showed a swelling in the right upper thigh that was hot and tender, but with no crepitus. He was unable to move his right hip, but had intact peripheral pulses.

Laboratory investigations showed a white blood cell count of 15000 per mm3, a hemoglobin level of 120 g/l, and a platelet count of 268 000 per mm3. He underwent hip and thigh X-rays [Figure 1] as well as CT scans of the abdomen and pelvis [Figure 2A & 2B].

**Figure 1:**
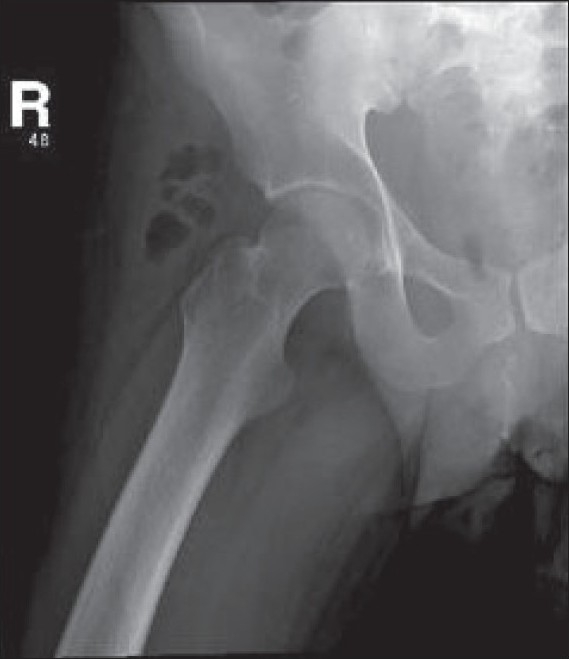
Plain X-ray of the right hip and thigh X-rays

**Figure 2A:**
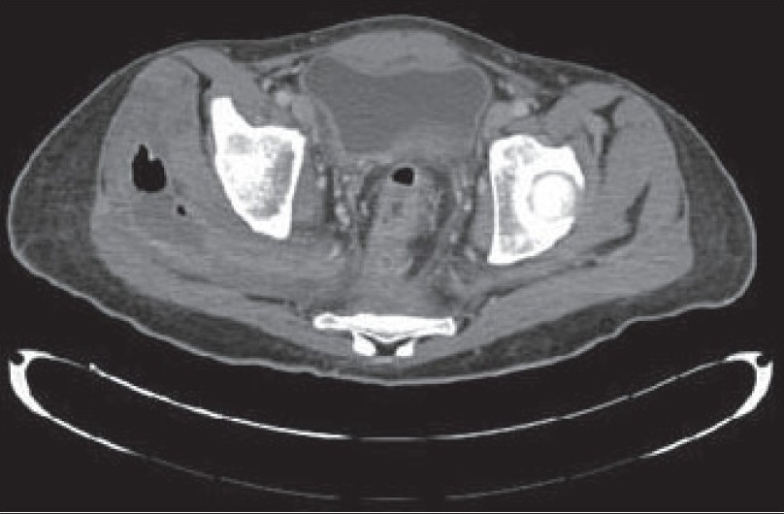
CT scans of the abdomen and pelvis

**Figure 2B:**
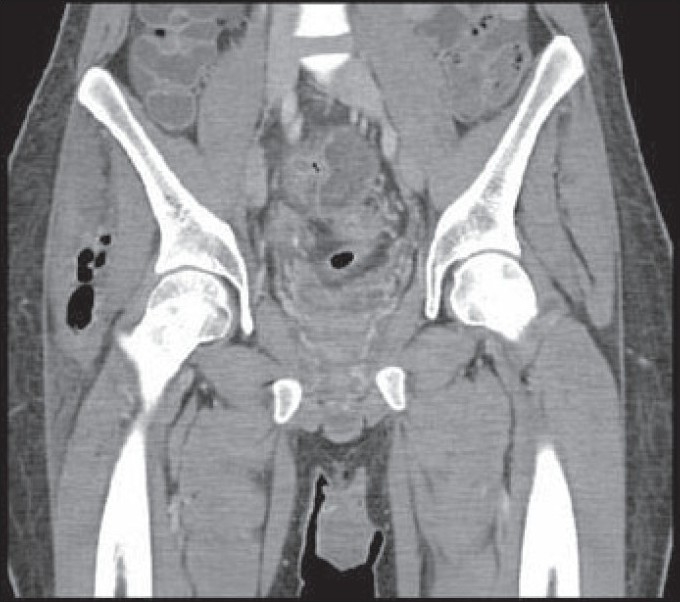
CT scans of the abdomen and pelvis coronal section

## QUESTIONS

1. Describe the radiological finding of figures 1, 2A and 2B?
2. What would be the most dangerous differential diagnosis?
3. What would be the appropriate management plan?

## ANSWERS

1. There are relatively large pockets of air seen in the soft tissue lateral to the right hip joint, which may indicate the formation of abscesses, probably caused by gas-forming organisms, or that have a fistulous communication with the bowel. Both hip joints are unremarkable.
2. Necrotizing fasciitis always needs to be considered and ruled out.
3. This patient needs i) urgent ICU admission, ii) hemodynamic resuscitation, iii) a septic screen, iv) broad-spectrum antibiotic coverage, v) laboratory investigations including muscle enzyme assays, vi) orthopedic and infectious disease teams to be involved, vii) diagnostically and therapeutically monitored drainage of the abscesses, in addition to special care and management of his Crohn's disease including viii) stool testing for culture and Gram staining, ova and parasites, and C. diffficle toxin and CMV antigenemia. At a later stage, his fistulizing disease needs to be evaluated with MRI of the abdomen and pelvis, and antiTNF therapy needs to be tried after ruling out sepsis. Colorectal surgeons need to be consulted if he does not respond.

